# Colorful 3D Reconstruction and an Extended Depth of Field for a Monocular Biological Microscope Using an Electrically Tunable Lens

**DOI:** 10.3390/biomimetics9010049

**Published:** 2024-01-12

**Authors:** Yang Cheng, Mengyao Liu, Yangqi Ou, Lin Liu, Qun Hao

**Affiliations:** 1 Key Laboratory of Biomimetic Robots and Systems, Ministry of Education, Beijing Institute of Technology, Beijing 100081, China; yangcheng2007@163.com (Y.C.); 18753293077@163.com (M.L.); ouyangqi0123@163.com (Y.O.); liulin_0530@126.com (L.L.); 2 Yangtze Delta Region Academy of Beijing Institute of Technology, Jiaxing 314003, China

**Keywords:** extended depth of field, color three-dimensional reconstruction, electrically tunable lens, monocular biological microscope

## Abstract

This paper presents a monocular biological microscope with colorful 3D reconstruction and an extended depth of field using an electrically tunable lens. It is based on a 4*f* optical system with an electrically tunable lens at the confocal plane. Rapid and extensive depth scanning while maintaining consistent magnification without mechanical movement is achieved. We propose an improved Laplacian operator that considers pixels in diagonal directions to provide enhanced fusion effects and obtain more details of the object. Accurate 3D reconstruction is achieved using the shape-from-focus method by tuning the focal power of the electrically tunable lens. We validate the proposed method by performing experiments on biological samples. The 3D reconstructed images obtained from the biological samples match the actual shrimp larvae and bee antenna samples. Two standard gauge blocks are used to evaluate the 3D reconstruction performance of the proposed method. The experimental results show that the extended depth of fields are 120 µm, 240 µm, and 1440 µm for shrimp larvae, bee tentacle samples, and gauge blocks, respectively. The maximum absolute errors are −39.9 μm and −30.6 μm for the first and second gauge blocks, which indicates 3D reconstruction deviations are 0.78% and 1.52%, respectively. Since the procedure does not require any custom hardware, it can be used to transform a biological microscope into one that effectively extends the depth of field and achieves highly accurate 3D reconstruction results, as long as the requirements are met. Such a microscope presents a broad range of applications, such as biological detection and microbiological diagnosis, where colorful 3D reconstruction and an extended depth of field are critical.

## 1. Introduction

The continuous development of microscopy technology has provided new tools and methods for bionics research. With the help of high-resolution biomicroscopes, researchers can observe and measure the microstructure and surface features of biological samples, which provide an important basis for their application to engineering and technology [1,2]. However, the depth of field of optical microscopy is always limited, resulting in clear images within the depth of field and blurred images beyond the depth of field. As a result, this shortcoming restricts complete observations of the object. Additionally, another significant drawback of traditional monocular biological microscopy is its constraint on the two-dimensional scale, which may not be as comprehensive and intuitive as three-dimensional (3D) observations. Up to now, numerous approaches have been proposed for reconstructing the 3D information from out-of-focus planes using a microscope [3,4,5]. Among them, there are two main methods for 3D reconstruction based on optical imaging, which are the stereo vision imaging method and the structured light method. The stereo vision imaging method employs two or more cameras to simultaneously capture images of biological samples from different viewpoints. By identifying similarities between these captured images that correspond to the same scene, a 3D reconstruction of the object can be achieved [6]. The main advantage of this method is that it can provide highly accurate 3D reconstruction results [7]. However, this method is sensitive to ambient light, and the simultaneous use of multiple cameras is both costly and bulky in size. Moreover, it increases the time consumption of data processing [8]. The structured light method adopts a light source to project a certain structural pattern on the surface of the sample to be measured, and the shape of the structural pattern is changed, and then the changed patterns on the object surface are observed by a camera to infer depth information of the sample [9]. The method has advantages in terms of efficiency and field of view [10]. However, it has high requirements for the light source because the secondary reflection of light often occurs, so the problem of light interaction produced by multiple light sources needs to be considered [11]. With the rapid development in the field of optical imaging, a 3D reconstruction method for the monocular biological microscope based on the shape-from-focus (SFF) approach has been developed [12,13]. By analyzing the relationship between object distance, focal length, and image sharpness using a combination of depth-of-field measurement and vertical scanning technology, the 3D information of the object can be recovered. The method has the advantages of low amount of calculation, high accuracy, and easy miniaturization [14,15]. The problem of the limited depth of field of the monocular biological microscope can be extended by switching a low-magnification objective or employing a translation stage to scan the sample in the optical axis direction to acquire multi-focus images [16,17]. Unfortunately, the switching of the objective or movement translation stage requires manual or mechanical operations, which makes the microscope complicated, bulky, complex, heavy, and expensive [18]. Moreover, manual or mechanical operation inevitably causes sample vibration that will affect the 3D reconstruction performance [19].

The electrically tunable lens (ETL) is a new type of lens proposed based on the principle of bionics, which mimics the structure of the human eye for fast and precise focus adjustment. Compared with the translation stage, the ETL can realize vibration-free axial scanning and is suitable for compact, fast-response, low-power microscopy [20,21,22]. In 2015, Jun Jiang et al. proposed a 3D temporal focusing microscopy using an ETL to extend the depth of field. The ETL provided a fast and compact way to perform non-mechanical *z*-direction scanning [23]. However, the magnification is changed when the focal length of the ETL changes, which will affect the imaging performance because the temporal focusing microscope is not a telecentric optical structure. In 2018, Yufu Qu et al. proposed monocular wide-field microscopy with extended depth of field to enable accurate 3D reconstruction [24]. However, the acquisition process and reconstruction algorithms are time-consuming because images from multiple views of the samples are required to acquire 3D point clouds to realize 3D reconstruction. In 2021, Gyanendra Sheoran et al. carried out a simulation and analysis of a combination of a variable numerical aperture wide-field microscope objective with an ETL for axial scanning with a telecentric image space [25]. However, it is difficult to place the ETL at the back focal plane of the objective for precise axial scanning with continuous resolution. Therefore, there is an urgent need for a microscope with extended depth of field and 3D reconstruction that can rapidly acquire and process images with accurate 3D reconstruction results for bionics research.

In this paper, we propose a biological microscope with colorful 3D reconstruction and extended depth of field using an ETL. To obtain high imaging performance, the magnification of the proposed microscope for extended depth of field is invariant, i.e., it is not appreciably affected by the focal length change in the ETL. It is realized by employing a telecentric 4*f* structure consisting of two identical relay lenses. The ETL is placed in the confocal plane of the 4*f* optical system and performs a continuous axial scanning of the sample without mechanical movement. By adjusting the focal length of the ETL, the images with different sharpness of the sample are obtained. Conventional biological microscopes encounter a paradox of high resolution and large depth of field. This optical limitation is overcome by using image fusion techniques to achieve both goals simultaneously. We propose to use an improved Laplace pyramid image fusion to expand the depth of field and thus present the key features of the sample. The 3D structure of the sample can be reconstructed using the SFF algorithm. During axial scanning, the state of each pixel in the image changes from defocus to focus to defocus. The sharpness of the pixel blocks in the image area is evaluated by the focus evaluation operator, and Gaussian curve fitting is performed on the evaluated values to obtain the depth information of each point to form a 3D depth map. We developed a monocular biological microscope prototype and carried out imaging experiments to verify its feasibility. Under the 10× objective, depths of field of 120 µm, 240 µm, and 1440 µm are obtained for the shrimp larvae, bee tentacle, and gauge block samples, respectively. The maximum absolute errors of the two standard gauge blocks are −39.9 μm and −30.6 μm, which indicates that 3D reconstruction deviations are 0.78% and 1.52%.

## 2. Optical Simulation of the 4*f* Optical System with an ETL

**Figure 1:**
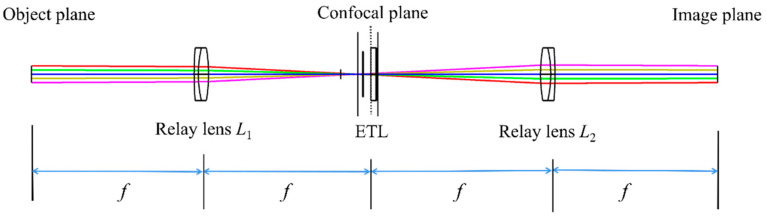
Schematic of the 4*f* optical system with an ETL.

If the monocular biological microscope using ETL is not telecentric, the change in the focal length of the ETL affects the magnification, which in turn affects the resolution and quality of the image. To achieve a large axial scanning range at constant magnification, the ETL needs to be placed at the back focal plane of the objective. However, the actual position of the back focal plane of the objective is usually within the barrel of the objective. Consequently, it is difficult to place the ETL exactly at the back focal plane of the objective. Fortunately, if the back focal plane of the objective is relayed out by two identical relay lenses forming a 4*f* configuration, the ETL can be placed at the conjugate plane of the back focal plane [26]. The 4*f* optical system is based on the Abbe imaging principle. It consists of two relay lenses (relay lens *L*_1_ and relay lens *L*_2_) with the same focal length, which cascades the front and back focal planes of the two relay lenses, as shown in Figure 1. Via the 4*f* optical system, an ETL can be easily placed at the confocal plane.

According to Fourier optics, the light field *f* (*x*, *y*) can be expanded into the superposition of countless complex functions.

(1) $$f(x,y)=\int_{-\infty }^{+\infty }\int_{-\infty }^{+\infty }F(f_{x},f_{y})e^{\left|i2\pi \cdot (f_{x}x+f_{y}y)\right|}df_{x}df_{y},$$

where *f_x_* and *f_y_* are the spatial frequencies in the *x* and *y* directions, respectively. *F*(*f_x_*, *f_y_*) is distributed as the spatial frequency spectrum with the variation in *f* (*x*, *y*).

The Fourier transform of *f* (*x*, *y*) can be expressed as

(2) $$F(fx,fy)=\int_{-\infty }^{+\infty }\int_{-\infty }^{+\infty }f(x,y)\cdot e^{\left|-i2\pi \cdot (f_{x}x+f_{y}y)\right|}dxdy,$$

In the 4*f* system, the object with the light field distribution *M* (*x*_1_, *y*_1_) is placed on the object plane and passed through the relay lens *L*_1_ to obtain the spectral function of the object. The light field distribution *M* (*f_x_*_1_, *f_y_*_1_) can be expressed as

(3) $$M(f_{x1},f_{y1})=F\{M(x_{1},y_{1})\},$$

The spatial spectrum of object *M* is obtained on the spectrum surface, the ETL is placed on the confocal plane, and after the transformation of the relay lens *L*_2_, the light field distribution *M* (*f_x_*_2_, *f_y_*_2_) is as follows:

(4) $$M(f_{x2},f_{y2})=F\{M(f_{x1},f_{y1})\}=FF\{M(x_{1},y_{1})\}=M(-x_{1},-y_{1}).$$

Thus, the image in the image plane is centrally symmetric with the image in the object plane. When the 4*f* optical system is added to the infinite remote microscope. The axial scanning range of the objective Δ*z* is as follows [5]:

(5) $$\Delta z=\frac{f_{r}\prime^{2}}{{M_{0}}^{2}f_{e}\prime },$$

where Δ*z* is the axial scanning range of the objective from the initial front focal plane, *f_r_′* is the focal length of the relay lens, *M*_0_ is the magnification of the objective lens, and *f_e_*′ is the focal length of the ETL. From Equation (5), it can be seen that the axial scanning range Δ*z* is proportional to the square of the focal length *f_r_*′ of the relay lens and inversely proportional to the focal length *f_e_*′ of the ETL and the square of the magnification *M*_0_ of the objective.

**Figure 2:**
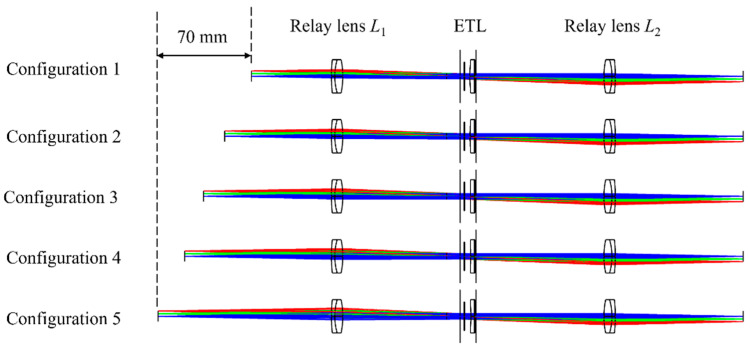
Ray tracing of multiple structures under five configurations when the focal length of the ETL changes from positive to negative.

Since the image of the object plane and the image plane are centrosymmetric, when the chief rays pass through the back focal point of the objective lens, they also pass through the center of the ETL because the position of the ETL is conjugated to the back focal plane of the objective lens. During the focus is scanned axially by changing the focal length of the ETL, the ETL does not change the propagation directions of the chief rays, and the image points corresponding to the chief rays are maintained. Hence, the magnification of the 4*f* optical system remains constant when the focal length of the ETL is altered [27,28]. To further verify the axial scanning function of the 4*f* optical system with an ETL, we perform an optical simulation using Zemax 19.4 software. To keep the magnification constant during axial scanning, the choice of the ETL position is key to maintaining the telecentric of the 4*f* optical system. In this simulation, the 4*f* optical system is constructed by two commercial relay lenses. Two achromatic lenses (#49-360, Edmund, NJ, USA) with a focal length of 100 mm are chosen as two relay lenses to minimize chromatic aberrations. A commercial ETL (EL-10-40-TC, Optotune, Dietikon, Switzerland) is placed in the confocal plane of the 4*f* optical system. To match the 10× objective lens with an *NA* of 0.25 used in the experimental section, the square space *NA* is set by 0.025 in the simulation. The ray tracing of multiple structures under five configurations of the 4*f* optical system with an ETL is shown in Figure 2.

**Figure 3:**
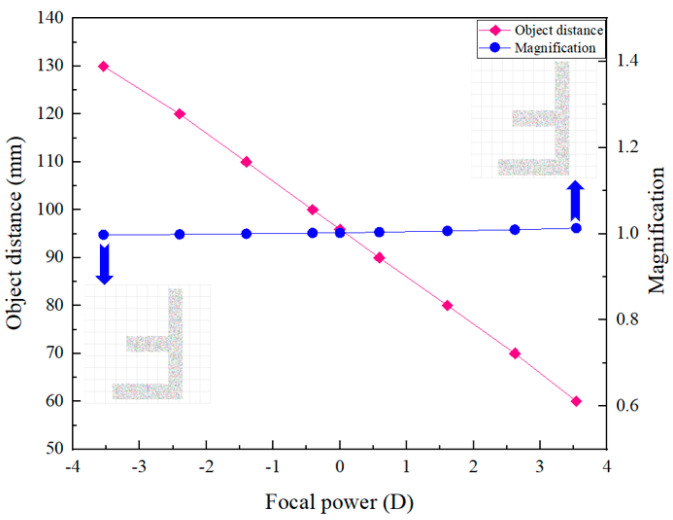
The relationship between the object distance, magnification of the 4*f* optical system, and the focal power of the ETL.

From Figure 2, we can see that by changing the focal length of the ETL, the axial scanning of the object can be achieved without moving the image plane. The relationship between the object distance and the focal power of the ETL is shown in Figure 3. From Figure 3, we can find that by adjusting the focal power of the ETL from negative to positive, the object distance shifts from 130 mm to 60 mm, corresponding to an axial scanning range of 70 mm. We also obtain the relationship between the magnification of the 4*f* optical system and the focal power of the ETL, as shown in Figure 3. It demonstrates that the object plane can be widely shifted by changing the focal length of the ETL without appreciably affecting the magnification of the 4*f* optical system. The maximum error of the magnification of the 4*f* optical system is 3.5%. From Figure 3, we can also find that when the focal powers of the ETL are −3.5 dpt and 3.5 dpt, the magnification of the simulated results of object *F* letter is invariant, i.e., is not appreciably affected by the focal power change in the ETL. Thus, when the ETL is located in the confocal plane, the 4*f* optical system becomes approximately telecentric, and the magnification remains constant.

**Figure 4:**
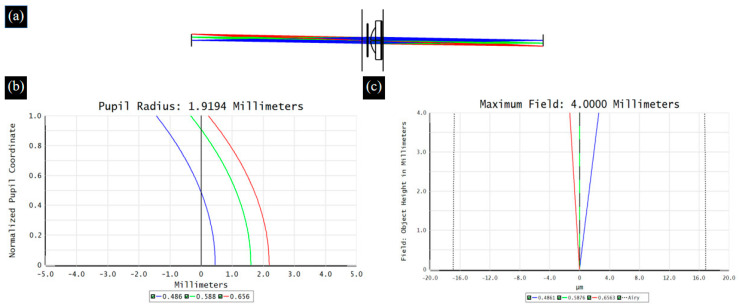
The optical path (**a**), axial chromatic aberration (**b**), and vertical chromatic aberration (**c**) results for the ETL.

**Figure 5:**
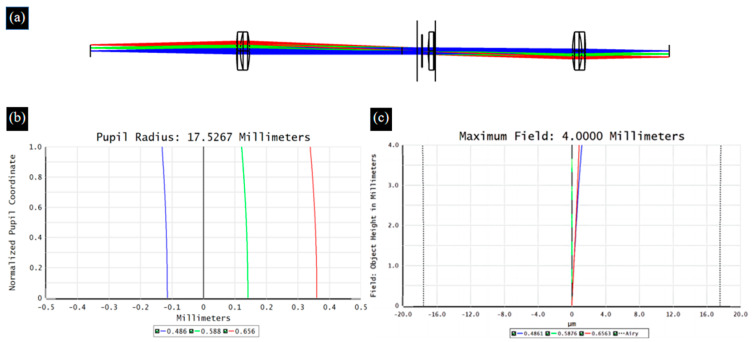
The optical path (**a**), axial chromatic aberration (**b**), and vertical chromatic aberration (**c**) results for the 4f system with an ETL.

When broad-spectrum light passes through an optical system, different wavelengths of light propagate along their respective optical paths, resulting in differences in imaging between wavelengths of light, which is defined as chromatic aberration [29]. ETL is a refractive optical element, and the dispersion characteristics of the material inevitably cause chromatic aberration problems. Therefore, in the proposed 4*f* optical system, we choose two sets of double-glued lenses to reduce the chromatic aberration of the system. Figure 4 and Figure 5 show the optical path, axial chromatic aberration, and vertical chromatic aberration results for the ETL only and the 4*f* system with an ETL, respectively.

## 3. Principle of the Laplace Pyramid Image Fusion

Limited by the depth of field, the microscope can only obtain clear images of the sample height within the depth of field. The images of the surfaces beyond the depth of field will become blurred. The calculation of the depth of field is shown as

(6) $$d_{DOF}=\frac{\lambda \cdot n}{NA^{2}}+\frac{n}{M\cdot NA}\cdot e,$$

where *d_DOF_* is the depth of field, *λ* is the wavelength of the illumination light, *n* is the refractive index of the medium between the sample and the objective lens, *NA* is the numerical aperture, *M* is the magnification, and *e* is the minimum resolvable distance. According to Equation (6), we can find that the depth of field decreases with increasing the magnification of the microscope. In the experiment, the depth of field of the 10× objective is 4.4 μm. To extend the depth of field, axial scanning of the object is performed by adjusting the focal length of the ETL in this paper. The images of the scanned sample at different focus positions are acquired. Via multi-focus image fusion technology, a fully focused image can be achieved, and accurate and complete image information can be obtained [30,31]. This technology compensates for the shortcomings of one source image and makes the details of the object clearer.

**Figure 6:**
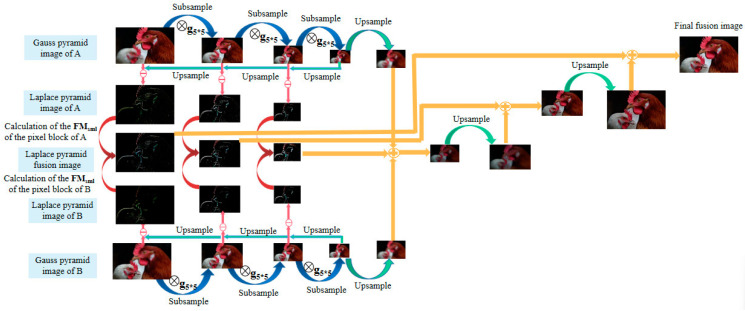
The flowchart of the improved Laplace pyramid image fusion method.

Microscopic imaging requires preserving as much detailed information as possible in the original image for analysis and processing. Laplace pyramid image fusion is capable of producing a series of images at different scales, which can be used to extract detailed information about the image. However, conventional fusion rules do not always produce optimal results for the focus region. Therefore, an improved fusion method of the Laplace pyramid is proposed in this paper. After the pyramid decomposition, the image forms a multi-scale map with different resolutions similar to the pyramid shape [32]. By comparing the source images at the corresponding scales, it is possible to extract the image details that are prominent in each of the source images into the fused image, enriching the fused image as much as possible in terms of the amount of information and achieving a better fusion effect. The flowchart of the improved Laplace pyramid image fusion method is shown in Figure 6.

Assuming that the original image is *A*, we make *G*_0_ (*i*,*j*) = *A* (*i*,*j*) (where 1 ≤ *i* ≤ *R*_0_, 1 ≤ j ≤ *C*_0_) as the initial layer of the Gaussian pyramid, namely layer 0, the first layer Gaussian pyramid can be generated by

(7) $$G_{k+1}(i,j)=\sum_{m=-2}^{2}\sum_{n=-2}^{2}s(m,n)G_{k}(2i+m,2j+n),(1\leq i\leq \frac{R_{k}}{2},1\leq j\leq \frac{C_{k}}{2}),$$

where *G_k_*(*i*,*j*) and *G_k_*_+1_(*i*,*j*) represent the image of the current layer and the image of the next layer, respectively, *R_k_* and *C_k_* represent the height and width of the image of the Gaussian pyramid of layer *k*, and *s*(*m*,*n*) represents the image mask to filter out the high-frequency part of the image.

The Gaussian pyramid is generated on the image, and the *k*+1th layer image of the Gaussian pyramid is *G_k+_*_1_. The Gaussian pyramid *G_k_*_+1_ of layer *k*+1 is convolved and interpolated to obtain *G*’*_k_*_+1_. The same arithmetic operation matches its size to the Gaussian pyramid *G_k_* of layer *k*. The gray values from *G_k_* to *G*’*_k_*_+1_ are subtracted, and the difference between the two adjacent layers of the image is obtained. The difference is usually the detail in the image processing. The Laplacian pyramid model is constructed from this difference in information.

(8) $${\{\begin{array}{l} LP_{k}=G_{k}-G_{k+1}^{\prime }=G_{k}-UP(G_{k})⊗g_{5*5},\;0\leq k<N\\ LP_{N}=G_{N},\;k=N \end{array}}_{,}$$

where *LP_k_* is the *k*th layer of Laplace’s pyramid. *LP_k_*, as the difference between *G_k_* and *G*’*_k_*_+1_, represents the information difference between two adjacent layers of pyramids, which is lost from the lower level of the pyramid. *UP* represents the upsampling of the image. *g*_5*5_ indicates the Gaussian convolution kernel with the window size of 5 × 5, which is represented as follows:

(9) $$g_{5*5}=\frac{1}{256}\left(\begin{array}{ccccc} 1 & 4 & 6 & 4 & 1\\ 4 & 16 & 24 & 16 & 4\\ 6 & 24 & 36 & 24 & 6\\ 4 & 16 & 24 & 16 & 4\\ 1 & 4 & 6 & 1 & 1 \end{array}\right).$$

Multi-source images have different features and details. Laplacian pyramid image fusion is used to filter these features and remove the blurred parts of the image using appropriate fusion rules to obtain a fully focused image [33]. For the fusion of Laplacian pyramids of the same level, a Gaussian pyramid is obtained by inverse Laplacian transformation, and the bottom image of the pyramid is the fused image. The traditional Laplace operator used for image fusion is

(10) ML(x,y)=|2I(x,y)−I(x−1,y)−I(x+1,y)|+|2I(x,y)−I(x,y−1)−I(x,y+1)|

(11) $$SML_{w1}(x,y)=\sum_{(i,j)\in w_{m\times n}}ML(x,y)\;ML(x,y)\geq T,$$

where *wn×n* is the size of the selected window and *T* is a threshold.

We performed image fusion according to the fusion rules of the modified Laplace operator (*MML*) and a multi-scale *SML*(*MSML*) to replace the traditional Laplace operator, as shown in the following:

(12) $$\begin{array}{ll} MML(x,y) & =\left|2I(x,y)-I(x-step,y)-I(x+step,y)\right|+\left|2I(x,y)-I(x,y-step)-I(x,y+step)\right|\\ & +\left|2I(x,y)-I(x+step,y-step)-I(x+step,y+step)\right|\\ & +\left|2I(x,y)-I(x-step,y-step)-I(x-step,y+step)\right|, \end{array}$$

(13) $$MSML(x,y)=\sqrt{{\left(SML_{W2}(x,y)-SML_{W1}(x,y)\right)}^{2}+{\left(SML_{W3}(x,y)-SML_{W1}(x,y)\right)}^{2}},$$

where the *step* represents the window size of the *ML* operator. *w*1, *w*2, and *w*3 are the three different sizes of the selected windows.

Compared with the traditional Laplace operator, the modified Laplace operator considers the change in clarity in the diagonal direction around the pixel points in the selected region; meanwhile, a variable spacing step to accommodate for possible variations in the size of texture elements is also added, so the judgment of the clear region is more robust and further improves the effect of the image fusion. Because a single window only considers a neighborhood of one scale, selecting a relatively small window is sensitive to noise, and selecting a relatively large window leads to overly smooth image fusion results. Therefore, a new multi-scale *SML* is used to take full advantage of different neighborhoods. By combining neighborhood information at different scales, the features and details of the image will be captured more comprehensively.

## 4. Principle of the Colorful 3D Reconstruction

**Figure 7:**
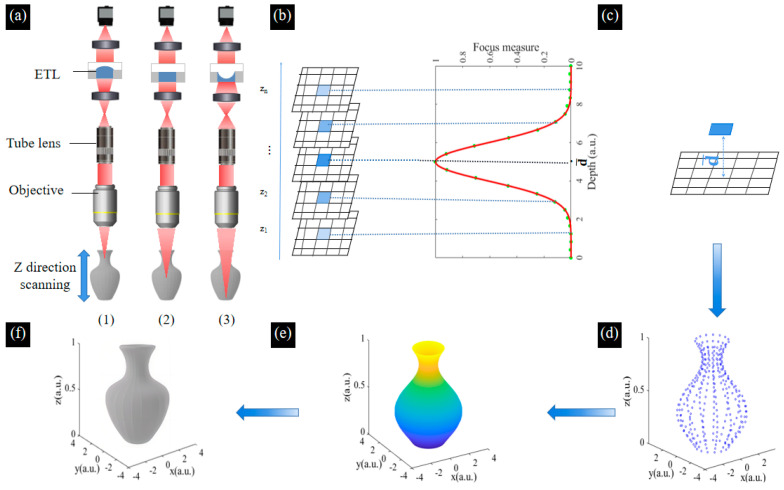
Schematic of the 3D reconstruction based on the SFF algorithm using the ETL. (**a**) Axial scanning for the sample using the ETL. The ETL presents as a (1) convex lens, (2) plate lens, (3) concave lens. (**b**) Focus measurements are performed using the clarity evaluation function and Gaussian curve fitting to obtain the height values. (**c**) Calculation of the depth of the selected point. (**d**) The discrete depth information of each point. (**e**) The depth map of the sample. (**f**) The colorful 3D reconstruction of the sample.

By mechanically moving the sample to obtain the image of different depths, the 3D information of biological samples can be solved. Unfortunately, this will cause the sample’s vibration, resulting in blurriness of the captured image which reduces the image resolution and affects the reconstruction accuracy [34]. In this paper, multi-focused images are obtained by changing the focal length of the ETL. The SFF algorithm is used to achieve the 3D reconstruction based on these multi-focused images, which reflects the relationship between the tested surface degree of focus and depth distribution. The focus measure (FM) function is used to extract depth information from the multi-focused image sequence. The 3D morphology of the tested sample surface is reconstructed according to the height information [35,36]. The schematic of the 3D reconstruction based on the SFF algorithm using the ETL is shown in Figure 7. Firstly, by varying the focal length of the ETL for axial scanning, sequential multi-focused images of the sample at different depths can be obtained. Each image has both clear and blurred focus regions, and each pixel of the image undergoes the process of defocus–focus–defocus in the image sequence, as shown in Figure 7a. Secondly, by defining a suitable window size for the image and evaluating the sharpness of the pixels in the image, we obtain the image sequence where the pixels with maximum sharpness are located, as shown in Figure 7b. Thirdly, based on the calibrated height at the location where the image sequence is taken, the depth value of the measured surface point corresponding to the pixel is obtained, as shown in Figure 7c. Lastly, the focus measurement operator measures the sharpness of the selected pixel block in the acquired sequence image. It is usually possible to directly select the position of the maximum value of the focus evaluation function curve as the depth value of a pixel point. Although it is possible to obtain a reconstructed image of the surface of the object, this leads to inaccuracies in the measurement because the image obtained is discrete, whereas the actual depth of the sample is continuous. Therefore, an interpolated fitting operation is required to obtain continuous depth information. In this paper, Gaussian curve fitting is used to obtain the height values close to the real surface microform. We obtain the height value of each point in the window to obtain the discrete depth information, which is interpolated and curve smoothed to obtain the 3D depth map, as shown in Figure 7d,e. The colorful information of the pixels in the image obtained by Laplace pyramid fusion is mapped to the corresponding positions in the depth map, as shown in Figure 7f. The process of 3D reconstruction of the image sequence is finally achieved.

Focus measurement is an important step in the process of 3D reconstruction and directly affects the accuracy of the 3D model. In this paper, the Tenengrad function is selected for focus measurement. The Tenengrad function calculates the gradient values horizontally and vertically by using the Sobel operator with the convolution operation for each pixel in the image. The two convolution kernels of the Sobel gradient operator are shown in Equation (14).

(14) $$\{\begin{array}{c} g_{x}=\frac{1}{4}\left[\begin{array}{ccc} -1 & 0 & 1\\ -2 & 0 & 2\\ -1 & 0 & 1 \end{array}\right]\\ g_{y}=\frac{1}{4}\left[\begin{array}{ccc} 1 & 2 & 1\\ 0 & 0 & 0\\ -1 & -2 & -1 \end{array}\right] \end{array},$$

The Tenengrad function based on the Sobel operator is calculated as follows

(15) $$F(I)=\{\begin{array}{ll} \sum_{x=2}^{M-1}{\sum_{y=2}^{N-1}\left[\nabla G(x,y)\right]}^{2}\; & \nabla G(x,y)>t\\ 0 & \nabla G(x,y)<t \end{array},$$

where *M* × *N* is the window size, and *t* is the threshold value introduced to modulate the sensitivity of the evaluation function. The Sobel Gradient operator ∇G(*x*, *y*) can be expressed as follows.

(16) $$\{\begin{array}{ll} \nabla G(x,y)= & \sqrt{\nabla G_{x}{\left(x,y\right)}^{2}+\nabla G_{y}{\left(x,y\right)}^{2}},\\ \nabla G_{x}(x,y)= & f(x+1,y-1)+2*f(x+1,y)+f(x+1,y+1)\\ & -f(x-1,y-1)-2*f(x-1,y)-f(x-1,y+1),\\ \nabla G_{y}(x,y)= & f(x-1,y+1)+2*f(x,y+1)+f(x-1,y+1)\\ & -f(x-1,y-1)-2*f(x,y-1)-f(x+1,y-1). \end{array}$$

The Tenengrad function is rotation invariant and isotropic, which can highlight the edges and lines in all directions, so it can be used as a criterion for the degree of image focus. In addition, the Tenengrad function uses the edge intensity for evaluating its sharpness and has high accuracy and certain anti-noise capability [37,38].

## 5. Experimental Results and Discussion

### 5.1. Experimental Setup

**Figure 8:**
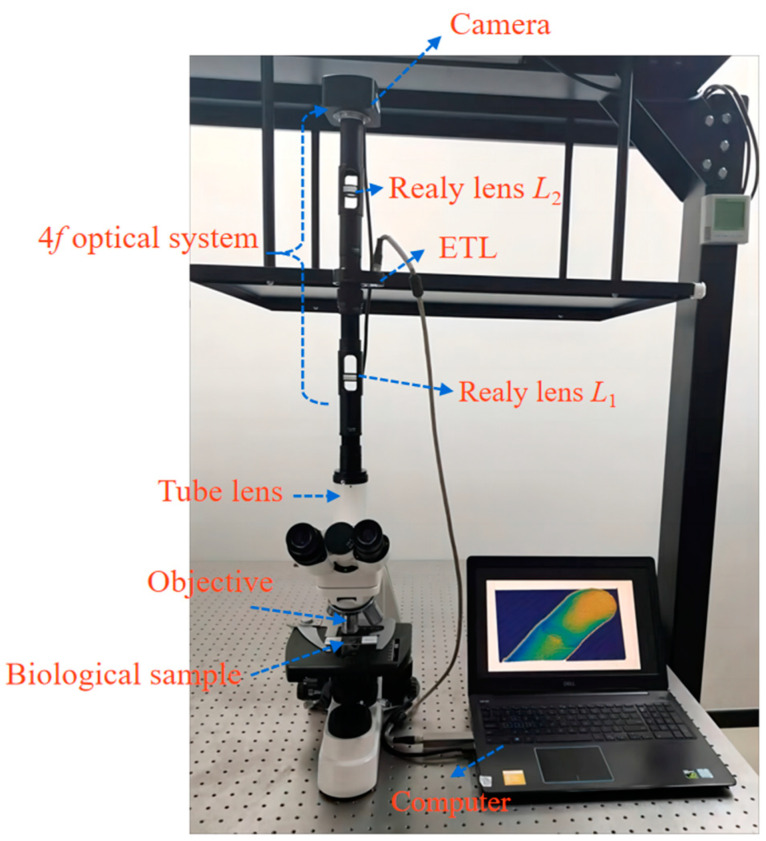
Experimental microscope setup of the extended depth of field and colorful 3D reconstruction using ETL.

The experimental setup mainly includes a monocular biological microscope (L208, AOSVI, Shenzhen, China), a 4*f* optical system, an ETL (EL-10-40-TC, Optotune), and a camera (3M180, AOSVI), as shown in Figure 8. An achromatic lens with a magnification of 10× and a numerical aperture of 0.25 functions as the objective. The biological sample is placed at the working distance of the objective. An LED light source with a wavelength from 400 nm to 760 nm is used for illumination. The 4*f* optical system includes two relay lenses with the same focal length of 100 mm and a diameter of 25 mm. The ETL can be tuned from a concave to a convex lens, resulting in a focal length range from −10 dpt to +10 dpt. Its response time is only 5 ms, which is faster than the translation stages. A camera with a resolution of 1632 × 1224 pixels is located in the image plane.

### 5.2. Experiment Analysis on Shrimp Larvae and Bee Antenna Samples

**Figure 9:**
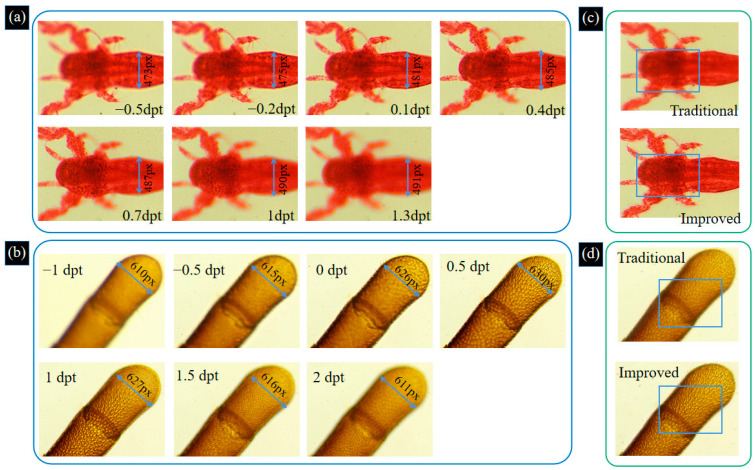
Image sequences of shrimp larvae (**a**) and bee antenna (**b**) samples are captured by adjusting the focal length of the ETL. The process of acquiring images of two biological samples by changing the focal power of the ETL is shown in Supplement S1. Traditional and improved Laplace pyramid image fusion result for shrimp larvae sample (**c**) and bee antenna sample (**d**).

To verify the effectiveness of the proposed system, experiments are carried out on biological samples. The shrimp larvae and bee antenna samples are observed under a 10× objective. By adjusting the focal power of the ETL, image sequences of these two samples are captured, as shown in Figure 9a,b. We analyze the image sequences of the shrimp larvae and bee tentacle samples and obtain that the maximum magnification errors of the two samples are 2.47% and 3.17%, respectively. The process of acquiring images of two biological samples by changing the focal power of the ETL is shown in Supplement S1. From Figure 9a,b, we can find that the full morphology of the sample cannot be focused on one image. Therefore, we use the improved Laplace pyramid image fusion method based on these image sequences to achieve the depth of field extension. We compare the improved Laplace pyramid image fusion algorithm with the traditional Laplace pyramid image fusion algorithm. The results of the two fusion methods are shown in Figure 9c,d.

To compare the fusion performance of the improved Laplace pyramid algorithm and the traditional Laplace pyramid algorithm, we choose the average gradient, the information entropy, and the standard deviation as the evaluation index. The average gradient reflects the contrast level of image details and texture change characteristics and reflects the contrast level of image details and texture change characteristics. Information entropy is mainly an objective evaluation index that measures the amount of information contained in an image. The higher the information entropy, the richer the information of the fusion image and the better the quality. The standard deviation is an objective evaluation index to measure the richness of image information. The larger the value, the more dispersed the gray level distribution of the image, the more information carried by the image, and the better the quality of the fusion image. The results of the fusion performance evaluation of the two fusion methods are shown in Table 1.

**Figure 10:**
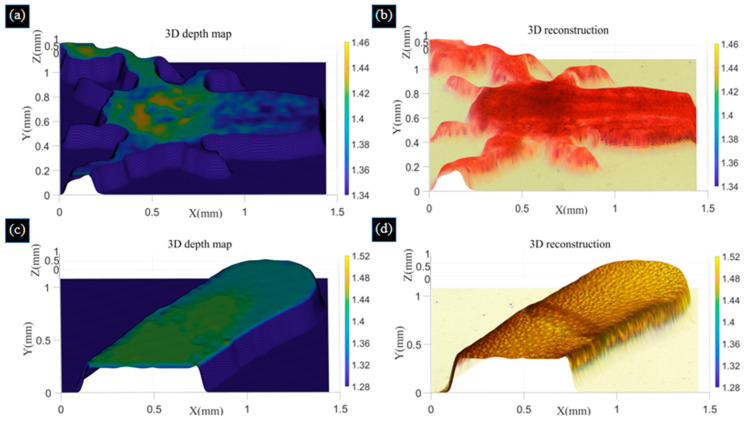
Depth map (**a**) and colorful 3D reconstruction (**b**) of shrimp larvae samples using the SFF algorithm. Depth map (**c**) and colorful 3D reconstruction (**d**) of bee antenna samples using the SFF algorithm.

From Table 1, the three evaluation indexes have been improved, indicating that the fusion images obtained with the improved Laplace pyramid image algorithm have more detailed details and better fusion effects. To further realize the 3D reconstruction of biological samples, we calibrate the gauge blocks to obtain the height of each image. We obtained the height of seven images as 1.28 mm, 1.32 mm, 1.36 mm, 1.40 mm, 1.44 mm, 1.48 mm, and 1.52 mm of the bee antenna samples, as well as the height of the shrimp larvae sample as 1.34 mm to 1.46 mm. We choose a window size of 5 × 5 to perform 3D morphology recovery using the SFF algorithm and use the Tenengrad function to obtain the depth map. The color information of the pixels in the image obtained by improved Laplace pyramid fusion is mapped to the corresponding positions in the depth map to obtain a colorful 3D reconstruction of the two samples, and the resulting images are shown in Figure 10. The reconstructed images we obtained match the actual bee antenna and shrimp larvae samples.

### 5.3. Three-Dimensional Reconstruction Performance Experiment on Gauge Blocks

**Figure 11:**
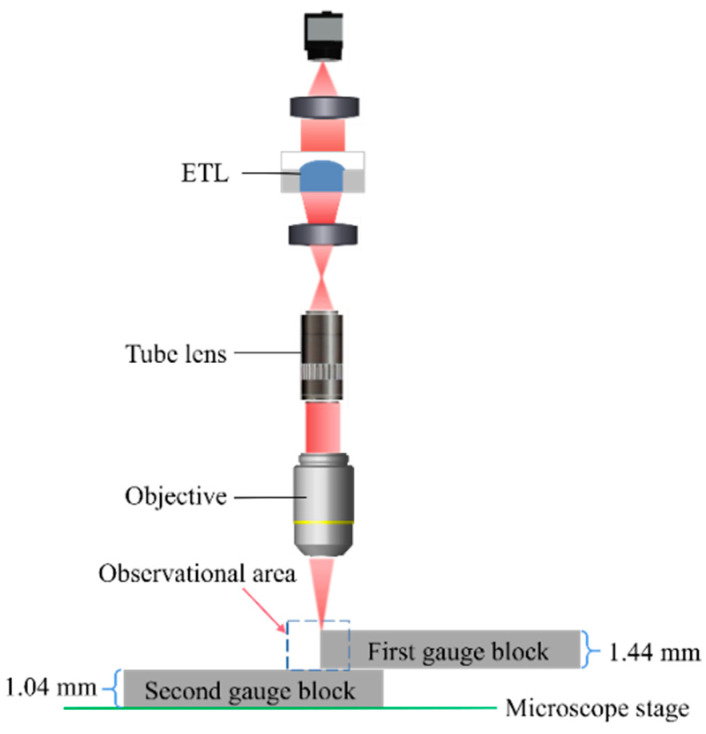
A schematic view of the experiment on two gauge blocks to evaluate the performance of 3D reconstruction.

To quantify the performance of the 3D reconstruction for the proposed microscope, two gauge blocks are stacked together to create a measurement interface that serves as a standard depth. In the experiment, the first gauge block with a height of 1.44 mm is placed on top of the second gauge block with a height of 1.04 mm under a 10× objective. Figure 11 shows a schematic view of the microscopic observation of the junction region. We adjust the ETL from −8.5 dpt to 7.5 dpt and take an image with a step of 2 dpt, giving nine images of the edge area of the gauge block. A schematic view of the experiment on two gauge blocks to evaluate the performance of 3D reconstruction is shown in Figure 11.

**Figure 12:**
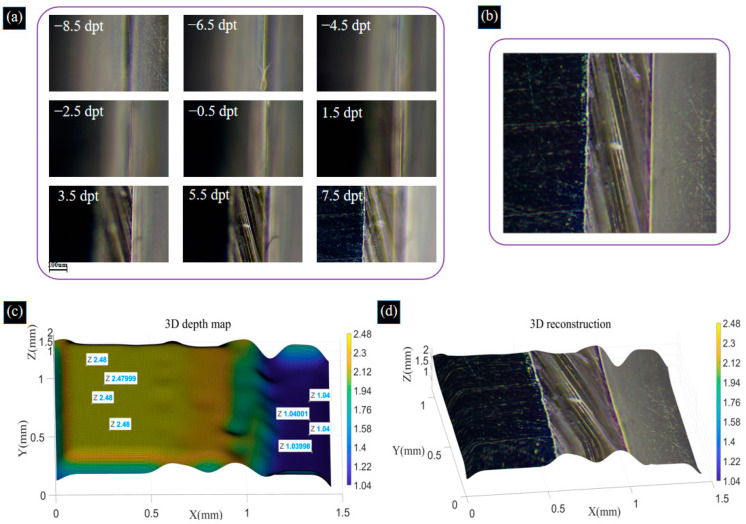
(**a**) Image sequences of gauge blocks captured by adjusting the focal power of the ETL. (**b**) Image fusion result for gauge blocks. (**c**) Recovered depth map of gauge blocks. (**d**) Colorful 3D reconstruction image of gauge blocks using the SFF algorithm. The process of acquiring images of gauge blocks by changing the focal power of the ETL is shown in Supplement S1.

Due to the narrow field of view under the high objective, the effective area of the two gauge blocks does not occupy the entire field of view, which has some impact on the reconstruction work. Therefore, we perform multiple measurements and 3D reconstruction processing and average the measurements to reduce the measurement error. The acquired image sequences of the two gauge blocks are shown in Figure 12a. The process of acquiring images of gauge blocks by changing the focal power of the ETL is shown in Supplement S1. The fusion image is shown in Figure 12b, the depth map is shown in Figure 12c, and the colorful 3D reconstruction map is shown in Figure 12d.

**Figure 13:**
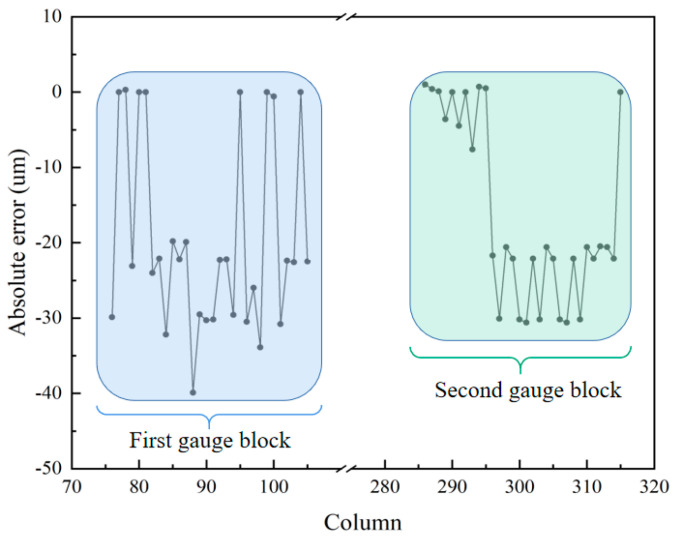
The absolute error of 3D reconstruction of the two gauge blocks.

In the 3D reconstruction image, 1050 data points are randomly chosen from columns 76 to 105 and columns 286 to 315 to calculate the deviation in the height of the two gauge blocks. Upon calculation, the average height of the first volume block is 2.4606 mm, and that of the second volume block is 1.0242 mm. The standard height of the first gauge block is 2.48 mm, and that of the second gauge block is 1.04 mm. The absolute errors of the gauge blocks are shown in Figure 13. From Figure 13, we can find that the maximum absolute errors are −39.9 μm and −30.6 μm for the first and second gauge blocks, respectively. The deviations in the reconstruction of both gauge blocks are 0.78% and 1.52%, respectively. Using a computer with an i5 core and 8 GM RAM, the acquisition and image reconstruction times for the shrimp larvae, bee antennae samples, and gauge blocks equal a total of 64 s, 72 s, and 84 s, respectively. This could be drastically improved by parallelizing the reconstruction process and using a graphics processing unit (GPU). In the proposed system, to achieve a large axial scanning range, we choose a relay lens with a focal length of 100 mm, resulting in a larger system size. In our future work, we will use a lens with a higher refractive index to reduce the size of the lens and, at the same time, choose a relay lens with a shorter focal length and optimize the distance between the optics to reduce the size of the overall system. These experiments demonstrate that the proposed system effectively extends the depth of field and achieves highly accurate 3D reconstruction results.

## 6. Conclusions

In this paper, we present a biological microscope with an extended depth of field and colorful 3D reconstruction using ETL. Fast axial scanning is achieved by changing the focal power of the ETL, which is located at the confocal plane of the telecentric 4*f* optical system. The optical simulation results show that the maximum error of the magnification change in the 4*f* optical system is 3.5%. In experiments on bee antennal and shrimp larvae samples, we have utilized the improved Laplace pyramid image fusion algorithm to improve on three evaluation metrics, including average gradient, information entropy, and standard deviation. The reconstructed images obtained from the biological samples are in good agreement with the actual bee antenna and shrimp larvae samples. Under the 10× objective, extended depths of field of 120 µm, 240 µm, and 1440 µm are obtained for the shrimp larvae, bee tentacle, and gauge block samples, respectively. In experiments on the junction region of the two gauge blocks, the maximum absolute errors of the two gauge blocks are −39.9 μm and −30.6 μm. The deviations of the 3D reconstruction are 0.78% and 1.52%. The experimental results show that the proposed microscopy can effectively extend the depth of field and obtain the colorful 3D reconstruction image of the samples using ETL. The proposed microscope has the potential to be used in 3D biological detection and microbiological diagnosis due to the advantages of an extended depth of field and high accuracy.

## Figures and Tables

**Table 1:** The evaluation index results of the two fusion methods for shrimp larvae and bee antenna samples.

	Evaluation Indicator	Fusion Algorithm	Average Gradient	Information Entropy	Standard Deviation
Sample					
Shrimp larvae		Traditional Laplace pyramid	3.764	6.987	64.992
		Improved Laplace pyramid	5.048	7.048	66.047
Bee antenna		Traditional Laplace pyramid	2.144	6.278	77.416
		Improved Laplace pyramid	3.671	6.435	78.838

## Data Availability

Data are available upon request due to privacy and ethical restrictions.

## Supplementary Materials

The following supporting information can be downloaded at www.mdpi.com/xxx/s1; Visualization S1: Experiment on samples by changing the focal power of the ETL.

## References

[1] Mahamdeh M., Simmert S., Luchniak A., Schaffer E., Howard J. (2018). Label-free high-speed wide-field imaging of single microtubules using interference reflection microscopy. J. Microsc..

[2] Jabbour J.M., Malik B.H., Olsovsky C., Cuenca R., Cheng S., Jo J.A., Cheng Y.S., Wright J.M., Maitland K.C. (2014). Optical axial scanning in confocal microscopy using an electrically tunable lens. Biomed. Opt. Express.

[3] Righetto R.D., Biyani N., Kowal J., Chami M., Stahlberg H. (2019). Retrieving high-resolution information from disordered 2D crystals by single-particle cryo-EM. Nat. Commun..

[4] Siegel N., Rosen J., Brooker G. (2012). Reconstruction of objects above and below the objective focal plane with dimensional fidelity by FINCH fluorescence microscopy. Opt. Express.

[5] Wouterlood F.G. (2014). 3D reconstruction of neurons from multichannel confocal laser scanning image series. Curr. Protoc. Neurosci..

[6] Machikhin A.S., Batshev V.I., Gorevoy A.V., Khokhlov D.D., Bykov A.A. (2019). A miniature prism-based stereoscopic system for 3D machine vision applications. Proc. SPIE.

[7] Su Z.L., Lu L., Yang F.J., He X.Y., Zhang D.S. (2020). Geometry constrained correlation adjustment for stereo reconstruction in 3D optical deformation measurements. Opt. Express.

[8] Kazmi W., Foix S., Alenyà G., Andersen H.J. (2014). Indoor and outdoor depth imaging of leaves with time-of-flight and stereo vision sensors: Analysis and comparison. ISPRS J. Photogramm. Remote Sens..

[9] Porras-Aguilar R., Falaggis K., Ramos-Garcia R. (2017). Optimum projection pattern generation for grey-level coded structured light illumination systems. Opt. Lasers Eng..

[10] Wen K., Fang X., Ma Y., Liu M., An S., Zhen J.J., Kozacki T., Gao P. (2022). Large-field structured illumination microscopy based on 2D grating and a spatial light modulator. Opt. Lett..

[11] Lachetta M., Sandmeyer H., Sandmeyer A., Esch J.S.A., Huser T., Müller M. (2021). Simulating digital micromirror devices for patterning coherent excitation light in structured illumination microscopy. Proc. Math. Phys. Eng. Sci..

[12] Ali U., Mahmood M.T. (2021). Robust focus volume regularization in shape from focus. IEEE T Image Process.

[13] Martišek D. (2018). Fast shape-from-focus method for 3D object reconstruction. Optik.

[14] Ute R., Sergey P. (2020). Application of the total focusing method for quantitative nondestructive testing of anisotropic welds with ultrasound. Tech. Mess.

[15] Nayar S.K., Nakagawa Y. (1994). Shape from focus. IEEE Trans. Pattern Anal. Mach. Intell..

[16] Yablon A.D. (2013). Multifocus tomographic algorithm for measuring optically thick specimens. Opt. Lett..

[17] Krauze W., Kus A., Sladowski D., Skrzypek E., Kujawinska M. (2018). Reconstruction method for extended depth-of-field optical diffraction tomography. Methods.

[18] Mutahira H., Muhammad M.S., Jaffar A., Choi T.S. (2013). Unorthodox approach toward microscopic shape from image focus using diffraction optical microscopy. Microsc. Res. Tech..

[19] Han M., Lei F.X., Shi W.J., Lu S.H., Li X.H. (2023). Uniaxial MEMS-based 3D reconstruction using pixel refinement. Opt. Express.

[20] Fahrbach F.O., Voigt F.F., Schmid B., Helmchen F., Huisken J. (2013). Rapid 3D light-sheet microscopy with a tunable lens. Opt. Express.

[21] Barak N., Kumari V., Sheoran G. (2020). Automated extended depth of focus digital holographic microscopy using electrically tunable lens. J. Opt..

[22] Ren H.R., Wu S.T. (2012). Introduction to Adaptive Lenses.

[23] Jiang J., Zhang D.P., Walker S., Gu C.L., Ke Y., Yung W.H., Chen S.C. (2015). Fast 3-D temporal focusing microscopy using an electrically tunable lens. Opt. Express.

[24] Qu Y.F., Zhang P., Hu Y.B. (2018). 3D measurements of micro-objects based on monocular wide-field optical microscopy with extended depth of field. Microsc. Res. Tech..

[25] Barak N., Kumari V., Sheoran G. (2021). Simulation and analysis of variable numerical aperture wide-field microscopy for telecentricity with constant resolution. Micron.

[26] Agour M., Fallorf C., Bergmann R.B. (2021). Fast 3D form measurement using a tunable lens profiler based on im-aging with LED illumination. Opt. Express.

[27] Qu Y.F., Hu Y.B. (2019). Analysis of axial scanning range and magnification variation in wide-field microscope for measurement using an electrically tunable lens. Microsc. Res. Tech..

[28] Kim J.W., Ahn J.S., Eom J.B., Lee B.H. (2019). Magnification-invariant surface profiling technique for structured illumination imaging and microscopy. Opt. Commun..

[29] Luo Z.Y., Li Y.N.Q., Semmen J., Rao Y., Wu S.T. (2023). Achromatic diffractive liquid-crystal optics for virtual reality displays. Light Sci. Appl..

[30] Li J., Sun Y. (2012). Image reconstruction algorithm for diffraction enhanced imaging-based computed tomography. Opt. Commun..

[31] Liu C., Jiang Z., Wang X., Zheng Y., Zheng Y.W., Wang Q.H. (2022). Continuous optical zoom microscope with an extended depth of field and 3D reconstruction. PhotoniX.

[32] Tian Y.Z., Hu H.J., Cui H.Y., Yang S.C., Qi J., Xu Z.M., Li L. (2017). Three-dimensional surface microtopography recovery from a multi-focus image sequence using an omnidirectional modified Laplacian operator with adaptive window size. Appl. Opt..

[33] Cheng Y.G., Zhu J.P., Hu S., Zhao L.X., Yan W., He Y., Jiang W.B., Liu J. (2017). Focusing properties of single-focus photon sieve. IEEE Photonics Technol. Lett..

[34] Kim C.S., Kim W., Lee K., Yoo H. (2019). High-speed color three-dimensional measurement based on parallel confocal detection with a focus tunable lens. Opt. Express.

[35] Fu B.Y., He R.Z., Yuan Y.L., Jia W.C., Yang S.C., Liu F. (2023). Shape from focus using gradient of focus measure curve. Opt. Lasers Eng..

[36] Zhang Z.Q., Liu F., Zhou Z.J., He Y., Fang H. (2021). Roughness measurement of leaf surface based on shape from focus. Plant Methods.

[37] Hu S., Li Z., Wang S., Ai M., Hu Q. (2020). Texture selection approach for cultural artifact 3d reconstruction considering both geometry and radiation quality. Remote Sens..

[38] Yang C.P., Chen M.H., Zhou F.F., Li W., Peng Z.M. (2020). Accurate and rapid auto-focus methods based on image quality assessment for telescope observation. Appl. Sci..

